# Bacterial-Derived Polymer Poly-γ-Glutamic Acid (γ-PGA)-Based Micro/Nanoparticles as a Delivery System for Antimicrobials and Other Biomedical Applications

**DOI:** 10.3390/ijms18020313

**Published:** 2017-02-02

**Authors:** Ibrahim R. Khalil, Alan T. H. Burns, Iza Radecka, Marek Kowalczuk, Tamara Khalaf, Grazyna Adamus, Brian Johnston, Martin P. Khechara

**Affiliations:** 1 Faculty of Science and Engineering, University of Wolverhampton, Wulfruna Street, Wolverhampton WV1 1LY, UK; Ibrahim.khalil@wlv.ac.uk (I.R.K.); A.T.Burns@wlv.ac.uk (A.T.H.B.); M.Kowalczuk@wlv.ac.uk (M.K.); Tamara.Khalaf@wlv.ac.uk (T.K.); B.Johnston@wlv.ac.uk (B.J.); 2 Polish Academy of Sciences, Centre of Polymer and Carbon Materials, Zabrze 41-819, Poland; Grazyna.Adamus@cmpw-pan.edu.pl

**Keywords:** γ-PGA, antimicrobial, drug delivery system

## Abstract

In the past decade, poly-γ-glutamic acid (γ-PGA)-based micro/nanoparticles have garnered remarkable attention as antimicrobial agents and for drug delivery, owing to their controlled and sustained-release properties, low toxicity, as well as biocompatibility with tissue and cells. γ-PGA is a naturally occurring biopolymer produced by several gram-positive bacteria that, due to its biodegradable, non-toxic and non-immunogenic properties, has been used successfully in the medical, food and wastewater industries. Moreover, its carboxylic group on the side chains can offer an attachment point to conjugate antimicrobial and various therapeutic agents, or to chemically modify the solubility of the biopolymer. The unique characteristics of γ-PGA have a promising future for medical and pharmaceutical applications. In the present review, the structure, properties and micro/nanoparticle preparation methods of γ-PGA and its derivatives are covered. Also, we have highlighted the impact of micro/nanoencapsulation or immobilisation of antimicrobial agents and various disease-related drugs on biodegradable γ-PGA micro/nanoparticles.

## 1. Introduction

**Figure 1:**
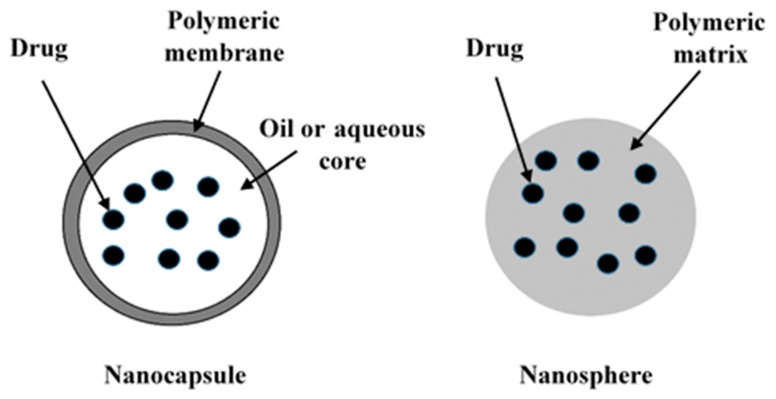
The two main types of polymeric nanoparticles are nanocapsules (reservoir system) and nanospheres (matrix system) with different drug-loading materials.

Nanoparticles (NPs) as drug delivery systems or direct anti-tumour systems facilitate unique approaches for many diseases. They have several benefits including: advances in detection, imaging, and delivering drugs to the site of infection. Liposomes, polymer-drug conjugates nanoparticles, polymeric nanoparticles and micelle nanoparticles are the most commonly used nanoparticles utilised in drug delivery systems [1,2]. Solid colloidal particles that are comprised of both nanocapsules and nanospheres are known as NPs. They are made through synthesis with preformed polymers and polymerisation methods [3,4]. The processes use minuscule droplets of liquid or solid materials that are coated with a polymeric material; this is known as nanoencapsulation. Nanocapsules are characterised by membrane-wall structures with an aqueous or oily core acting as a reservoir to contain the bioactive material (Figure 1). In contrast, nanospheres exhibit matrix systems where the bioactive material is dispersed throughout the particles [5]. The main characteristic feature of nanoparticles is their size, which can be 5–10 nm with 1000 nm as an upper size limit. However, the range of use is usually 100–500 nm [6]. As emphasised by various authors, these nanoparticulated drug delivery systems are known for their great capacity as active vectors that can control and sustain the release of drugs at a site of localisation [4]. Their advantage of higher intracellular uptake over many other particulate systems is due to their subcellular size, and the ability to improve the stability of active substances [7]. They are also biocompatible with cells and tissue when made from materials that are biodegradable and biocompatible [8,9]. Moreover, nanoparticulated drug delivery systems have several other advantages over other drug delivery systems; this includes the fact that drugs can be incorporated without any chemical reactions, they stabilise drug activity, the drugs themselves are protected against degrading agents such as light and pH, there is a decrease in tissue irritation and high efficiency of drug encapsulation [10,11]. Researchers have extensively studied polymeric micro/nanoparticles as drug carriers in the field of pharmaceuticals [12,13,14,15]. Additionally, different researchers have published reviews on the mechanism of nanoparticle formation, the organisation of nanoparticulated systems and the preparation techniques of nanoparticles [6,15,16,17,18]. Nanoparticles that are biodegradable are usually used in the improvement of the therapeutical value of many water-insoluble and soluble drugs and molecules that are bioactive through the enhancement of solubility, bioavailability and retention time [19,20]. 

A very promising approach for developing nanomedicine is through the nanoencapsulation of the small molecules or the drugs in nanocarriers. This conventional drug entrapment technique gives efficient drug loading inside the nanocarriers, thus reducing the toxicity linked with the drugs. The nanocarrier targeting can enhance the effectiveness of the nanoencapsulated drug at the disease site [21]. The nanoparticle’s performance in vivo is usually influenced by surface chemistry, molecular weight and morphological characteristics. Nanoparticles that are surface modified have the property of being anti-adhesive through the extended arrangement of the particle surface. Acting as a steric barrier it decreases the degree of clearance by circulating macrophages of the liver and endorses the likelihood of undergoing improved permeation processes [19,20]. The release mechanism can be controlled through selection of the molecular weight of the polymer used. The higher the molecular weight of a polymer, the slower the in vitro drug will be released. Some of the problems challenged by new classes of active molecules can be solved by the careful design of delivery systems with respect to the route and target of administration [22]. Another application of nanoparticles is the directing of organs or tumours to increase their selective cellular internalisation and binding by receptor-mediated endocytosis. Targeting ligands are usually grafted to the nanoparticle’s surface via a linkage [23]. Moreover, confidence in nanotechnology success is high, given numerous applications including textiles, agriculture, fibre, forensic science, medical therapies and electronics [19,24].

The most prevalent synthetic polymers for micro/nanoencapsulation are, poly (lactic-co-glycolic acid) (PLGA), poly(glycolic acid) (PGA) and poly(lactic acid) (PLA). They have all been approved by the US Food and Drug Administration (FDA) for particular medical applications, as they are biodegradable and are easily removed from the body in the form of water and carbon dioxide molecules [22,25]. However, these polymers may reduce the surrounding pH during polymer degradation, which subsequently affects cellular function by creating a highly acidic microenviroment, thus limiting their application in vivo [26]. Increased attention has turned to the use of biopolymers to overcome this problem. Naturally occurring biopolymers produced by living organisms during cell growth may be the key, as they can be produced in the laboratory and they show noticeable synthesising flexibility. One of the most recently discovered biopolymers for the application of nanoparticles is poly gamma glutamic acid (γ-PGA). Attention has been drawn to γ-PGA because it is biodegradable, biocompatible, non-toxic and non-immunogenic. Few reviews exist thus far that have explored γ-PGA designed nanoparticle drug delivery systems, which are used in many biomedical applications including vaccination, inflammation, cancer and other diseases [27,28,29]. In this review, the fabrication and characterisation techniques of γ-PGA nanoparticles and its derivatives are covered. In addition, we have highlighted the impact of nanoencapsulation and the immobilisation of various disease-related drugs with biodegradable γ-PGA nanoparticles.

## 2. Poly-γ-Glutamic Acid (γ-PGA) as an Antimicrobial Coating and Drug Delivery System

Naturally occurring poly-γ-glutamic acid (γ-PGA) is a biopolymer, which is biodegradable, water soluble, non-immunogenic and non-toxic for humans and the environment. It consists of monomer units of d-glutamic acid or l-glutamic acid or both. Glutamate is polymerised inside the cell through the γ-amide linkages. The polymer α-PGA is produced by chemical synthesis, while γ-PGA is produced microbiologically by a number of *Bacillus* species [27,29,30,31,32]. γ-PGA can be degraded in vivo to glutamic acid residues. The polymer is also secreted extracellularly as a free polymer into a fermentation medium, in fed-batch cultures. *Bacillus licheniformis* ATCC 9945a can produce γ-PGA with yields of more than 30 g/L. *B. licheniformis* and *B. subtilis* are characterised as safe microorganisms that, through cultivation, produce microbial γ-PGA [29,33,34]. Ashiuchi et al., 2015 observed that γ-PGA ion-complexes (PGAICs) maintained surface antimicrobial activity. The complex of γ-PGA and a compound used in toothpaste called hexadecyl-pyridinium (HDP) is suitable as an anti-fungal agent due to its coating stability [35]. In order to control the release of bound and encapsulated drugs, in drug delivery applications, polymers of different molecular sizes are needed. Polydispersity and molecular weight determine the properties and applications of a polymer [4,36,37]. During chemical synthesis, the molecular weight of α-PGA can be controlled; γ-PGA’s molecular weight is dependent on the source of its production. Alkaline hydrolysis used to vary the molecular weight of microbial γ-PGA, other applications including ultrasonic degradation, microbial or enzymatic degradation and alteration of medium composition have also been tried [30,38,39]. 

For nanoparticulate delivery systems, drugs are usually encapsulated in the nanoparticle’s polymer matrix or core. In recent years, there has been tremendous interest in polymeric nanoparticles for pharmaceutical and biomedical applications because of their in vivo biocompatibility, sub-micron size, and their precise and constant releasing properties [40,41]. The development of new biomaterials and their use as drug delivery carriers has resulted in the progression and alteration of new and current methods for the fabrication of nanoparticles. Attention has been drawn to γ-PGA because of its diverse properties, as already mentioned. Because of these properties, γ-PGA designed nanoparticles are used as drug delivery systems for many biomedical applications including cancer therapy, antimicrobial gene therapy, biological adhesives, vaccines and other applications [27,30].

### 2.1. Method of γ-PGA Nano/Microparticle Formulation

Delivery systems are particulates designed for the regular release of pharmaceutical agents in order to maintain therapeutic drug levels for an appropriate period of time in the body. This is attained through the encapsulation of these therapeutic agents into a polymer particle that is degradable, and the continuous release of the agent as the polymer matrix degrades [42,43]. Drug delivery systems (DDS) with nanoparticles are promising since they reduce toxic side effects and enhance the improvement of the desired therapeutic effect. The choice of NP preparation method is based on a number of factors such as the type of polymeric system, the area of application, size requirements, surface charge, molecular weight, hydrophobicity, hydrophilicity, biodegradability, biocompatibility and solubility of the drugs. For example, NPs that are produced for application in the medical field ideally should be totally free from additives or reactants [17,18]. Polymers that are block copolymers or self-assembled polymers have the ability to form nanostructures, that have been investigated in the fields of pharmaceuticals and biotechnology. Overall electrostatic forces, van der Waal forces, hydrophobic interactions, and hydrogen bonding are considered the key forces leading to the formation of the polymer [44,45]. There are a few γ-PGA-based nanoparticle preparation methods, and each differs in their organisation structure. The active molecule or a specific drug is usually included inside the centre of the nanocapsule, or it is absorbed into the matrix surface, of the nanosphere. The self-assembling polymer or block/graft copolymer method and the ionic gelation method are the most common methods for γ-PGA nanoparticle formulation [27]. To date, all of the formulated γ-PGA particle studies have been in nanoscale size, apart from three studies which investigated the fabrication of γ-PGA particles in micro scale size [46,47,48].

#### 2.1.1. Solvent Exchange Method

**Figure 2:**
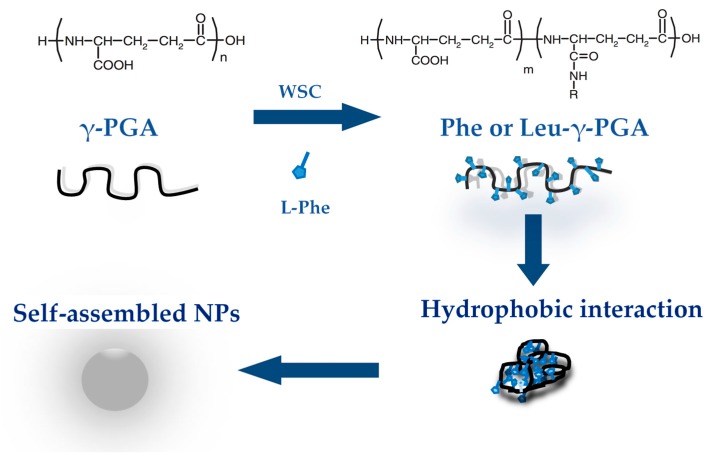
Chemical structures of poly-γ-glutamic acid (γ-PGA) and γ-PGA hydrophobic derivatives achieved in the presence of water-soluble carbodimide (WSC), and the subsequent formation of self-assembled nanoparticles (NPs). Adapted from Matsusaki et al., 2004 [51] and Piyapakom, 2014 [52].

Nanoparticles that are made by the self-assembly of copolymers that are amphiphilic blocks or ones that are hydrophobically manipulated show success as drug carriers [49]. Generally, the copolymers made from hydrophilic or hydrophobic sections are able to form structures that are polymeric in aqueous solutions through hydrophobic interactions. These nanoparticles are made from inner hydrophobic moieties and outer hydrophilic shells [15,50]. Matsusaki et al., 2004 reported the first formation of bacterial γ-PGA nanoparticles [51]. They used γ-PGA as the hydrophobic backbone and l-phenylalanine (Phe) or l-leucine methyl esters (Leu) as the hydrophobic sections. l-phenylalanine or l-leucine methyl esters were grafted to γ-PGA in the presence of the water-soluble carbodimide (WSC) (Figure 2). These γ-PGA-Phe or γ-PGA-Leu copolymers with 30%–58% grafting degrees (determined by NMR) formed nanoparticles that were monodispersed in water because of their amphiphilic characteristics.

In order to make self-assembled nanoparticles by the solvent exchange method, 10 mg/mL of γ-PGA-Phe was dissolved in DMSO that was then added to the same amount of distilled water. The particles were then dialysed to remove DMSO and then lyophilised. The average size of the γ-PGA-Phe NPs was approximately 200 nm, whereas γ-PGA-Leu NPs showed submicron order aggregations [51]. Kim et al., 2009 [53] found that the size of the γ-PGA-Phe formed monodispersed nanoparticles that can be easily controlled. By altering the NaCl concentration in distilled water, different NP sizes were obtained and ranged from 30 nm to 200 nm. Due to the ionisation of the carboxyl group found near to the γ-PGA surface, the nanoparticles showed highly negative charges, and the zeta potential was −25 mV. In the same study, it was shown that another hydrophobic amino acid l-tryptophan methylester (Trp) can be grafted to γ-PGA in order to form self-assembled nanoparticles. Nanoparticles were obtained using the precipitation and dialysis methods. However, the authors found that γ-PGA-Trp NPs could not be redispersed in phosphate buffer saline (PBS) after lyophilisation by freeze drying. All sizes of γ-PGA-Trp NPs aggregated and formed relatively large NPs when dispersed in PBS. In contrast, freeze-dried γ-PGA-Phe NPs redispersed well in PBS and the size distribution was the same as before lyophilisation. It is believed that the difference in dispersibility of freeze-dried γ-PGA NPs in PBS may be due to the difference in hydrophobicity between Trp and Phe. Moreover, the shape of γ-PGA-Trp NPs was more uniform and spherical than γ-PGA-Phe particles, yet γ-PGA-Trp NPs had a soft and flexible morphology. The size of particles ranged from 34 to 192 nm and 58 to 292 nm in γ-PGA-Phe NPs and γ-PGA-Trp NPs, respectively, depending on the increase in the concentration of NaCl. No evidence was found for the comparison of drug encapsulation efficiency and release profiles between γ-PGA-Phe NPs and γ-PGA-Trp NPs [53].

For drug encapsulation, γ-PGA loaded with protein nanoparticles were made, about 0.25–4 mg of bovine serum albumin was dissolved in 1 mL of saline buffer, followed by the addition of 1 mL of γ-PGA-Phe to the protein solution. Subsequently, the solution that resulted was centrifuged and rinsed constantly. Overall, the encapsulation of proteins with different molecular weights and isoelectric points into the nanoparticles was successful [54,55]. The efficiency of the encapsulation ranged between 30% to 60% in most samples. The encapsulation effectiveness for each protein was found not to be influenced by the protein’s physical properties. Moreover, the nanoparticle size increased when proteins were encapsulated within it. The increase for albumin, when it was encapsulated, increased the nanoparticle’s size, from 180 nm to 256 nm. This could be due to the swelling capacity increasing because of the hydrophilic properties of the proteins in the nanoparticles [27]. All the nanoparticles could be preserved through freeze-drying. The cytotoxicity test results for the nanoparticles have shown that they do not lead to cell damage. Furthermore, studies have proven that γ-PGA-Phe nanoparticles could have broad potential in the future as carriers with multiple functions for delivering drug and vaccines in biomedical and pharmaceutical applications [53,54]. However, the full properties of association are still not completely clear, and the protein-loaded structure requires further research.

#### 2.1.2. Ionic Gelation Method

**Figure 3:**
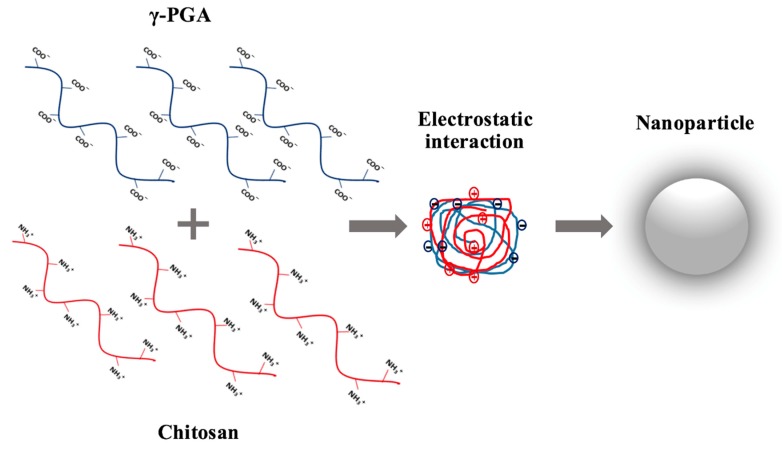
Development of polyion complex (PIC) nanoparticles comprised of polyelectrolytes that are oppositely charged, giving electrostatic interactions. As shown, γ-PGA is the negatively charged polymer, while the positively charged polymer is Chitosan.

The ionic gelation technique uses surface-charge contact between a polymer and a polyanion/polycation or two oppositely charged polymers [56,57]. Lin et al., 2005 [58,59] used γ-PGA and Chitosan (CH) to form Polyion complex (PIC) particles in aqueous media (Figure 3). They found that different concentrations of CH (0.01%–0.2%) and γ-PGA produced smooth-surfaced and spherical nanoparticles of different zeta potentials and particle sizes (140–370 nm). Nanoparticles were made by the addition of negatively charged γ-PGA (160 kDa at pH 7.4) into positively charged CH (50 kDa at pH 6.0) under magnetic stirring. Using ultracentrifugation nanoparticles were then collected. Following the zeta potential, the particle size of the γ-PGA and CH nanoparticles were determined; mainly through the quantity of the γ-PGA concentration found in the solutions added to the concentration of CH in the surrounding media. When CH is at a fixed concentration, an increase in γ-PGA allows the γ-PGA molecules to interact with the CH, resulting in the formation of bigger nanoparticles. When the number of CH molecules are more than γ-PGA molecules, the extra CH molecules are located on the surface of the γ-PGA-CH nanoparticle [58,59,60]. When a number of CH molecules exceeded that of local γ-PGA molecules, some of the excessive CH molecules were located on the γ-PGA-CH nanoparticle surfaces. The formed nanoparticles could have a neutral PIC core structure with a positively charged CH surrounding the shell in order to confirm colloidal balance. The research indicates that the nanoparticle size ranges from 80 nm to 400 nm with a surface charge ranging from −35 to +25 mV of γ-PGA-CH that can be controlled by altering the ratio of the mixture used in the two polymers [61].

In PIC nanoparticles, the key stability parameter is the pH and the ionic strength; this is due to the electrostatic interactions being influenced by the shielding of the ionic species [62]. Under physiological conditions, the weakening of the PIC limits their use as drug carriers. When the γ-PGA-CH NPs are at pH 7.4, a deprotonation of the CH occurred, which led to the collapse or aggregation of nanoparticles. The nanoparticles that remained were in the range of pH at 2.5–6.6. To overcome this, nanoparticles that are multi-ion-cross-linked were made with γ-PGA and CH mixed with Tripolyphosphate (TTP) and MgSO_4_, and they showed significant increases regarding their stability [63]. These cross-linked nanoparticles have a greater constancy over a wider pH range than the nanoparticles that are uncross-linked [27]; in order to increase the microparticle’s stability in a very broad range of pH, this was done through the use of magnesium sulfate and the TTP. They were utilised for the physical linking of γ-PGA to CH by ionic gelation which is being used increasingly in the field of biomedical science.

### 2.2. Nanoparticle Characterisation Techniques

**Figure 4:**
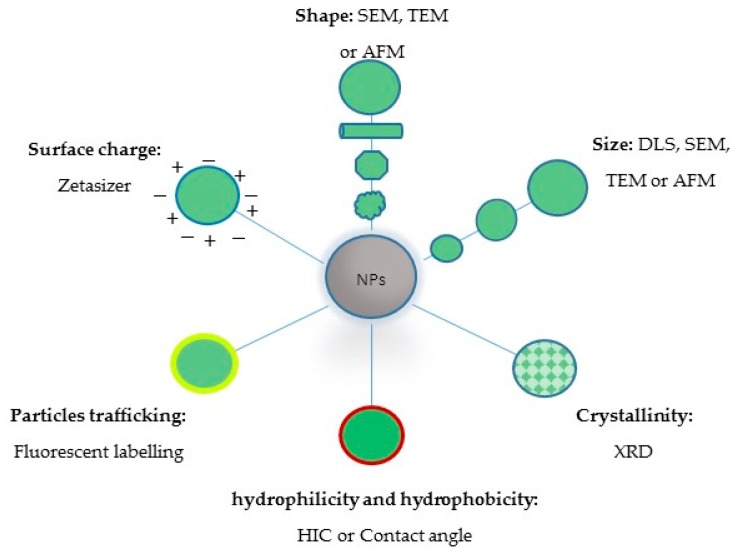
Essential techniques for nanoparticles (NPs) characterisation; SEM: scanning electron microscopy; TEM: transmission electron microscopy; AFM: atomic force microscopy; DLS: dynamic light scattering; XDR: X-ray diffraction; HIC: hydrophobic interaction chromatography.

To understand the properties before developing them for additional medical application, nanoparticle characterisation is vital (Figure 4). The size of nanoparticles is very important for several reasons: to determine the degradation rate, the release profile and to establish the effectiveness of the therapeutic agent with regard to cellular uptake and tissue penetration [64]. The size distribution, morphology and particle size are determined by scanning electron microscopy (SEM), transmission electron microscopy (TEM), atomic force microscopy (AFM) or mainly by dynamic light scattering (DLS) based on photon correlation spectroscopy [65,66]. The physical form of the polymer and the drug must be known, as this will influence the in vivo and in vitro drug release properties. Nanoparticles’ longevity and intracellular trafficking of particles as a function of pH and mucoadhesion are all affected by the zeta potential. Furthermore, the nanoparticle distribution in the body following administration is determined by hydrophobicity. Hydrophilic particles tend to stay in the blood for a lengthier time [66,67,68]. A Zetasizer is widely used to determine the zeta potential. The zeta potential results can be negative or positive, and they are determined by the type of polymer or the materials used for surface modification. This method is commonly employed to identify the nanoparticle’s surface charges [46,69]. In addition, hydrophilicity and hydrophobicity are determined via the corresponding hydrophobic interaction chromatography (HIC) or water contact angle measurements [70]. NPs which are usually used for drug delivery in an amorphous phase are more preferable than the crystalline phase, as the last is not accessible in general. The crystallinity of NPs is determined by X-ray diffraction (XRD) [71]. The encapsulation efficiency, degradation rate and nanoparticle size of the polymer are influenced by the molecular weight of the polymer. Polymer chain length is correlated with the molecular weight, where the higher the molecular weight, the longer the length of the chain. Additionally, the length of the chain reveals the lipophilicity or hydrophilicity of the polymer. When the chain length is increased, it decreases the degradation rate of the polymer and raises its lipophilicity. Thus, through varying its molecular weight, the degradation rate of the polymer and the kinetic release of the drug can be controlled. Size exclusion chromatography usually determines molecular weight [41,72,73].

## 3. Medical Applications of γ-PGA-Based Micro/Nanoparticles

The polymer γ-PGA is expected to be a useful material-drug conjugate in the future. Nanoparticles made from natural or artificial polymers have applications in many biomedical and technological fields because of their surface functionalities, chemical structures and their controlled size. Nanoparticles containing therapeutic agents that have been entrapped successfully include peptides, proteins and various small molecules previously discovered to have major potential as drug delivery systems [74,75]. Here, we are detailing the use of γ-PGA-based micro/nanoparticles as a delivery vector to treat different diseases. Table 1 displays the summary of γ-PGA-based micro/nanoparticles drug delivery applications with particle size and encapsulation efficiency (EE%).

### 3.1. Antimicrobial Therapy

Antimicrobial peptides (AMPs) are promising antibiotics as they present a wide range of antimicrobial activity against gram-negative and gram-positive bacteria, parasites, enveloped viruses and fungi. These antibiotics simply do not induce resistance (in contrast to other antibiotics). These peptides are the only human peptides from the cathelicidin family that present high antibacterial activities; this is due to the biological properties the molecule possesses. Many studies involving the AMP encapsulation and biopolymer immobilisation led to improving the active agent’s release [98]. High concentrations of the peptide for an extended time period should be maintained to achieve optimal therapeutic effects sufficiently. For this, Sun et al., 2015 revealed a novel microencapsulation system loaded with AMPs made from poly-γ-glutamic acid and CH and prepared through the ionic gelation process [46].

A study by Hoennscheidt et al., 2013 [99] has found that the drug quinine was encapsulated partially with γ-PGA of a high molecular weight made from *B. licheniformis*. This drug (Quinine) is known to be the most popular anti-malarial drug linked with very severe side effects when overdosed. This drug is not effectively water-soluble; it works in a prototypical manner to many hydrophobic drugs. Steady distributions were made through the use of a technique based on solvent evaporation and other characteristics [99]. For acquired immunodeficiency syndrome (AIDS) treatment, the drug Saquinavir (SQV) is used as a protease inhibitor. However, it has disadvantages, including low permeability through the blood-brain barrier in clinical practice [100]. Usually, the blood-brain barrier has a high transendothelial resistance to many substances such as the antiretroviral drugs from entering the brain through the paracellular route [101]; this makes transcellular transport the main pathway for the delivery of SQV to the central nervous system. Brain microvascular endothelial cells are the major component of the blood-brain barrier; they have sialic acid residues of glycoproteins which are acidic thus aiding in the production of an anionic domain on the luminal front of the cell. These endothelial cells have the ability to attract entities that are positively charged, through electrostatic interaction and the use of conjugation and cationic colloids has led to the enhancement of the effectiveness of antiretroviral SQV and endocytosis [100,101].

Kuo et al., 2011 [100,102] conducted a study that aimed at developing nanoparticles made with SQV-polylactide-co-glycolide (PLGA) at the core of the nanoparticle and γ-PGA and Polyethyleneimine (PEI) on the surface which was used as a system for drug delivery. The hydrophobic PLGA nanoparticles can spread and aid the dissolution properties of SQV from the PEI/γ-PGA/SQV-PLGA nanoparticles through the hydrophilic γ-PGA and the grafted PEI charge. The study investigated the size distribution, the morphology of the particle and the efficiency of the PEI grafting in addition to the effectiveness of the entrapment of the SQV and the release of SQV kinetics. To release and encapsulate SQV, the PEI/γ-PGA/SQV-PLGA nanoparticles were produced. The entrapment efficiency of the SQV could be improved by using SQV at low concentrations. Moreover, PEI at a high concentration delayed the employment of free PEI on to the surface medium of γ-PGA/SQV-PLGA nanoparticles. PEI at high grafting competence causes the underdevelopment of SQV from the PEI/γ-PGA/SQV-PLGA nanoparticles [100,102]. 

### 3.2. Cancer Therapy

Cancer is one of the most lethal diseases and the chemotherapy used to treat it is known to be very toxic towards healthy tissues. The most recognised and used anticancer drugs are known to have a short half-life in plasma with limited solubility in aqueous conditions, which reduces their efficiency as therapeutic drugs [93,103]. A study was developed by Liang et al., 2006 [93] on the formulation of poly (γ-glutamic acid)-poly (lactide) nanoparticles filled with paclitaxel in order to treat liver cancer. Paclitaxel is commonly used to treat breast and ovarian cancer; however, it can also be used with many other types of cancer as many cancer cells such as hepatoma cells are killed when exposed to this drug [93,104]. To target liver cancer cells, the group introduced glucosamine to Paclitaxel-loaded nanoparticles conjugated to the carboxyl (–COO–) groups on γ-PGA to work as a moiety. Asialoglycoprotein receptors found on the surface of hepatoma cells enables them to recognise the *N*- and the galactose acetylgalactosamine-terminated glycoproteins (ASGP). The formulated particles used were about 128 nm with a zeta potential of 19.6 mV. The study concluded that the complexes have the ability to target hepatoma tumour sites through the ASGP receptor-affected recognition, leading to a reduction in tumour size. Therefore, they are capable of being used as a system for drug delivery to both liver cancers and other liver conditions [104].

Cancer drugs are usually directed into the body as intravenous infusions that may cause a burst-like release and decay of the drug concentration in the blood. Successfully sustained drug release formulations have shown that long-term exposure of ailing tissues to a reasonable drug concentration is more useful and better than a higher concentration of the drug [28]. Manocha and Margaritis, 2010 [28] have successfully made an ionic complex NP containing Doxorubicin/γ-PGA. Doxorubicin (DOX) is an anthracycline antibiotic commonly used as an antineoplastic mediator; the association of DOX on nanoparticles was shown to have controlled release over an extended period of time, therefore increasing its effectiveness and also reducing the toxic side effects that come with it. The carboxyl groups offer an attachment point for the chemotherapeutic drugs to the γ-PGA carrier [85,105]. The study aimed to investigate the use of the carboxyl group with the γ-PGA polymer, in order to optimise the formation of ionic complexes with anticancer cationic drugs. The specific connections between DOX and γ-PGA, to produce robust ionic complexes, were also investigated. It was found that carboxyl groups have the ability to produce strong ionic connections with the selected drugs because they are great hydrogen bond donors. The finding may act to help provide a new drug carrier for the controlled release of DOX in malignant tissues. The particle size was between 80 to 86 nm with a zeta potential of −30 to −56 mV [86].

Another study, Matsuo et al., 2010 [80] discovered that γ-PGA NPs are excellent delivery carriers for antigens and for stimulate production of antigen-loaded dendritic cells (DCs) used in many cancer immunotherapies. Vaccine systems based on dendritic cells through the use of γ-PGA NPs as a delivery system for antigen carriers appear to have many applications in the design of vaccines aimed to trigger the responses of T cells, helpful in cancer therapy and developing chronic viral disease vaccines [80].

### 3.3. Gene Therapy

In gene therapy, there are three different methods applied for gene delivery. The first one is the use of naked DNA, at high levels of gene expression, by the direct injection of free DNA to the infected site. This appears to be suitable for treating tumour sites that are readily available by direct injection, like skin and muscles, but it is unsuitable for a systemic delivery. The second approach involves using genetically altered viruses. These viruses in viral vectors have the ability to transfer their genetic materials into the host cells [106]. The third method of delivery is the use of non-viral vectors that have a cationic nature, provided by cationic polymers or cationic lipids. They interact with DNA through electrostatic interactions, as DNA is negatively charged, leading to the formation of polyplexes and lipoplexes, respectively [107].

Currently the most important approach in gene therapy via a systemic pathway is the development of non-toxic gene vectors that are stable, that can easily encapsulate and that are capable of treating specific cell types such as cancerous cells by delivering foreign genetic materials [107]. Yet complexes based on NPs lead to many problems regarding the release of DNA when it arrives at the required site, making its transfection efficiency rather limited. In order to develop effective vectors to be used for DNA transfection, knowledge of the internalisation mechanism is vital. To solve this, a study carried out by Peng et al., 2009 [85] developed and modified the internal structure of CH/DNA complexes through the incorporation of negatively charged γ-PGA. The possible internalisation mechanism of CH/DNA/γ-PGA complexes was investigated through the use of transmission electron microscopy and the inhibitors specific to different endocytic pathways [85]. Complexes of CH/DNA were taken up by pathways mediated by micropinocytosis and caveolae (sub-microscopic plasma membrane pits that are available in many mammalian cells). Both pathways were enhanced through the incorporation of γ-PGA in the DNA/CH complex, yet the caveolae-mediated pathway played the most important role. Following internalisation, the amount of CH/DNA/γ-PGA entering the lysosomes was less than that CH/DNA alone, which led to enhanced expression at the gene level [85].

High-specificity Small Interfering RNAs (siRNAs) are known to silence target genes; they therefore have the potential of being a new strategy to provide therapeutic drugs that lower unwanted gene expression. Yet there are many disadvantages concerning the use of siRNA for therapy, including degradation in the intracellular cytosol and plasma, leading to a shorter half-life. Moreover, the internalising of naked siRNAs cannot be done efficiently inside cells [108]. A carrier system based on CH with the addition of γ-PGA for a siRNA delivery was conducted by Liao et al., 2010 [96]. The results obtained from this study revealed that γ-PGA has a vital role in the enhancement of the cellular uptake of CH complexes: providing increased siRNA release, leading to an improvement in gene silencing and extending the therapy’s duration. Combining the complexes of CH, siRNA and γ-PGA presents an innovative method that can be used as a proficient siRNA transfection vector [96].

### 3.4. Diabetes Therapy

Drugs being administrated orally are preferable since it is painless and straightforward. Medication that is orally administrated would be particularly valuable in the management of many diseases, especially chronic ones like diabetes that need medication over a lifetime [109,110]. There are many issues regarding patient compliance, because of the inconvenience of injections on a daily basis in addition to the insulin therapy that is given by a subcutaneous injection. Thus orally administrated insulin is better as it enters the portal circulation where it goes via the liver before it reaches systemic circulation, allowing it to resemble the insulin secreted physiologically, in comparison to the well-known conventional administration by subcutaneous procedures that may lead to peripheral hyperinsulinemia [63,89]. Oral insulin administration will, therefore, benefit patients greatly. However, in the gastrointestinal (GI) tract, insulin is known to be inherently unstable as it has a low penetrability for intestinal epithelium [111,112]. In order to provide an effective insulin delivery system, nanoparticles should have balanced characteristics; including their size, which needs to be large enough to carry insulin but also small enough to permit uptake and diffusion. Mucoadhesion extends the housing time of nanoparticles during their absorption, although it can cause a delay in their diffusion through the mucous layer of the GI tract. Many nanoparticle formulations have been presented and tested, many of which have been modified in order to find the exact configuration so that they can be used for oral insulin delivery [112].

Several studies formulated an insulin delivery system composed of CH/γ-PGA nanoparticles [89,90,91,92]. These NPs were sensitive to pH, which led to an insulin absorption increase in the intestine, as the γ-PGA NPs increased the insulin residence time in the small intestine, which led it to infiltrate better into the mucus layer, and then mediate the transient opening of the junction between enterocytes where the insulin was released by the pH sensitive NPs. In diabetic rats the results showed moderate, extended hypoglycemia with a bioavailability of about 15%; however, this increased to 20% when the nanoparticles were enteric-coated capsules. Nanoparticles that are cross-linked with multi-ions were formed when γ-PGA was mixed with CH, MgSO_4_ and tripolyphosphate. Such complexes are considered to be stable and more compact over a range of pH values, in addition to being more effective in insulin transport than simple nanoparticles. Insulin being transported through the use of CH γ-PGA nanoparticles was shown to be an effective alternative to the usual application of subcutaneous basal-acting insulin [90].

The study presented by Sonaje et al., 2010 [89] discussed the use of nanoparticles that are pH sensitive, with γ-PGA/CH shielding providing an oral administration system for insulin through a paracellular pathway. Chitosan can adhere to the surface of epithelial cells, leading to the transient openings in the junctions of contiguous cells [89]. The study also reported the use of enteric-coated capsules with the freeze-dried nanoparticles to be used for the oral delivery of insulin. The main idea of the enteric-coated capsule is for it to remain intact in the stomach, within an acidic environment and to begin dissolving when it reaches the small intestine, which has a neutral environment. This encapsulation may lead to the prevention of the disintegration of the nanoparticles in the stomach, thereby leading to an increase in the overall number of nanoparticles being presented to the small intestine, consequently increasing the bioavailability of insulin [89]. 

### 3.5. Vaccine Therapy

Vaccines are used as an active device in the modulation of host immune responses to target a specific antigen, particularly microbial and pathogen-associated antigens [113]. The immunogenicity of vaccines is enhanced by the use of adjuvants through the use of various methods. Without the utilisation of an adjuvant, the effective immune response induced by a vaccine often fails. Many studies are in place to evaluate the vaccine adjuvant and antigen delivery systems based on particles, designed to achieve very effective immune responses [114]. Nanoparticles made from poly-γ-glutamic acid and l-phenylalanine ethyl ester have resulted in cellular and humoral immune responses that are antigen-specific for a wide range of antigens. γ-PGA nanoparticles may exhibit more significant antigen-specific adaptive responses in laboratory animals than simple antigens or antigens mixed with additional adjuvants. γ-PGA nanoparticles carrying antigens have led to effective anticancer and antiviral immunity, demonstrating fully their importance as capable vaccine delivery system [83].

A study conducted by Uto et al., 2013 [83] revealed that γ-PGA nanoparticles serve as antigen carriers, endorse dendritic cell maturation and encourage the presentation of antigens through class I major histocompatibility compex (MHC) molecules. Such nanoparticles stimulate rapid antigen-specific cluster of differentiation 8 (CD8) T cell activation. γ-PGA nanoparticles efficiently provoke an adaptive immune response by dendritic cell arbitrated in in vivo immunity, which could lead to them being used as antigen delivery systems [83].

These authors developed an efficient single-dose immunisation procedure through the administering of Japanese encephalitis virus-like particles (JE-VLP) with γ-PGA nanoparticles. They showed that specific cell-mediated and humoral immune reactions associated with JEV were improved through the addition of adjuvants. When injected into a mouse, the γ-PGA nanoparticles did not provoke any injury to the tissue or penetrate inflammatory cells. Moreover, they did not lead to any acute toxicity of the cells. Using a two-dose procedure, the γ-PGA nanoparticles were directed into the mouse, where they did not lead to any anti-γ-PGA nanoparticle immune responses [76]. Overall, the study showed that one dose of JE-VLP combined with γ-PGA nanoparticles provoked antibodies that are JE-specific, thereby enhancing defence responses against the virus. Based on this study, the third generation of JE vaccines are to be designed and could be used on military workers and travellers [76]. The outcome of such studies is awaited with great interest.

### 3.6. Gastroesophageal Reflux Disease (GERD)

Lansoprazole sodium works as a proton pump inhibitor which decreases the secretion of gastric acid. With decreasing pH, the degradation rate and the instability of lansoprazole is increased [47]. In the stomach, the degradation of lansoprazole occurs because of to the acidic environment, leading to a reduction in clinical effect and bioavailability. However, lansoprazole microparticles within enteric capsules display better absorption characteristics than enteric tablets. A microparticle method made from poly glutamic acid and chitosan was established for oral lansoprazole delivery through the use of an ionic gelation method. In the GI tract, the pH environment is different, as the stomach has an acidic environment and the small intestine has an alkaline environment. In order to increase the stability of microparticles, a compact structure of carriers was developed to work in a wider range of pH, sodium tripolyphosphate and magnesium were introduced to crosslink CH physically with γ-PGA in such investigations [47,89,90].

The TTP and the sulphate salts are routinely cross-linked via ionic gelation in many biomedical applications. The FDA have recognised sodium tripolyphosphate as a food additive. Between carboxylate ions on γ-PGA and Mg^2+^ ions, physical gelation might occur through electrostatic interactions. The most abundant ion in living cells is Mg^2+^. Singh et al., 2012 [47] prepared microparticles that are pH-sensitive, and subsequently complexed with CH, TTP and MgSO_4_, and utilised with γ-PGA in different ratios. The research concluded that microparticles are free flowing with a round shape; many chitosan properties were altered with the addition of γ-PGA, leaving γ-PGA-CH micro/nanoparticles, a very useful drug delivery application carrier. Acid-dependent drugs, including lansoprazole, can now be expressed in the form of an enteric covered capsule with chitosan/γ-poly-glutamic acid microparticles in them, used as a continued drug release delivery method [47,89,90].

## 4. Conclusions

Delivering macromolecules and small molecular weight drugs for localised or targeted delivery to specific sites has been a challenging task in the past. The development, improvement and advances in nanotechnology-based drug delivery systems have offered appropriate new solutions for this kind of application. Poly-γ-glutamic acid (γ-PGA) is a very promising non-toxic, non-immunogenic and biodegradable polymer that can be produced naturally by various strains of *Bacillus*. Different roles of γ-PGA such as a biocontrol agent, cryoprotectant, probiotic protectant, bacteriophage protectant or thickeners have been reported. This review is meant to contribute to the understanding of γ-PGA nanoparticles’ (NPs) methods of fabrication and trace the development of γ-PGA nanoparticles and their medical applications in treating different diseases. Techniques such as ionic gelation are straightforward and do not involve any organic solvents, harsh chemicals or mechanical treatments. Using fermentation techniques, γ-PGA can be produced simply and extracellularly with high yields. Using nanoparticles as drug delivery systems based on nanoparticle γ-PGA, as detailed in this review, leads to the prediction that they will be crucial in the future due to their unique properties and safety features. However, the detailed characterisation of the in vivo performances of these well-designed nanoparticles is lacking. More systematic studies must be performed to regulate the performance and consistency of these nanoparticles in the blood or lymphatic fluids. Much more in-depth study is vital for understanding the details of how drugs are released when γ-PGA-based nanoparticle vectors enter target cells.

## Figures and Tables

**Table 1:** Poly-γ-glutamic acid (γ-PGA)-based micro/nanoparticles drug delivery applications.

Drug Loaded in PGA Micro/Nanoparticles	Applications	Size (nm)	EE%	Reference
Adjuvant	Japanese encephalitis virus vaccine	200	-	[76]
Antigen	Vaccine development	150–300	55–60	[49,53,54,77,78,79,80,81,82,83]
DNA	Gene therapy	130–204	94–99	[84,85]
Doxorubicin	Antineoplastic therapy	150–630	51–69	[86]
Erythromycin	Antimicrobial therapy	220–280	43–80	[87]
Fibroblast growth factor and heparin	Human foreskin fibroblast cells	206–272	85–97	[88]
Insulin (oral)	Diabetes therapy	241–6120	44–73	[89,90,91,92]
Lansoprazole	Gastroesophageal reflux disease	342–367 μm	75–92	[47]
Paclitaxel	Liver cancers	128	50–53	[93]
Peptides (LL-37) and nitric oxide	Antimicrobial therapy	793–2128	13–76	[46]
Proteins such as ovalbumin	Tumour vaccines	250	55–60	[94,95]
RNA	Gene Therapy	216	-	[96]
Saquinavir	Antiretroviral therapy	188–307	77	[97]
